# Telomerase Reverse Transcriptase (TERT) Mutation at a Price: Telomere Biology Disorder Presenting With Pancytopenia and Cirrhosis Requiring Liver Transplantation

**DOI:** 10.7759/cureus.116042

**Published:** 2026-09-10

**Authors:** Taha Fadlou Allah, Marc M Kesselman, Sahar S Amini, Sonia Daryanani, Anjali Bhasin

**Affiliations:** 1 Medicine, Nova Southeastern University Dr. Kiran C. Patel College of Osteopathic Medicine, Davie, USA; 2 Rheumatology, Nova Southeastern University Dr. Kiran C. Patel College of Osteopathic Medicine, Davie, USA; 3 Internal Medicine, Nova Southeastern University Dr. Kiran C. Patel College of Osteopathic Medicine, Davie, USA

**Keywords:** cirrhosis, danazol, liver transplantation, pancytopenia, telomere biology disorder, tert mutation

## Abstract

Telomere biology disorders (TBDs) are inherited conditions that can present in adulthood with pancytopenia and liver disease. We describe a case of a 45-year-old man with a high body mass index (BMI), long-standing macrocytic pancytopenia, and biopsy-proven steatohepatitis who was found to have markedly short telomeres and a heterozygous telomerase reverse transcriptase (TERT)variant. The patient had a clear hematologic response to danazol but developed progressive cirrhosis with portal hypertension, ultimately requiring orthotopic liver transplantation with sleeve gastrectomy; after transplant, liver chemistries normalized, but cytopenias recurred under tacrolimus-based immunosuppression. This case highlights the need to consider TBDs in adults with unexplained macrocytic cytopenias and progressive liver disease, and to recognize that liver transplantation corrects hepatic failure but not the underlying bone marrow disorder.

## Introduction

Telomeres are repetitive DNA-protein structures at chromosome ends that preserve genomic stability by protecting against degradation and end-to-end fusion; progressive telomere shortening triggers cellular senescence or apoptosis and contributes to age-related organ dysfunction [1,2]. Telomere biology disorders (TBDs) are caused by germline variants in genes involved in telomere maintenance, including the telomerase reverse transcriptase (TERT) gene, and can affect multiple organ systems, with manifestations including bone marrow failure, pulmonary fibrosis, and liver cirrhosis [3]. TERT encodes the catalytic reverse transcriptase component of telomerase, which is essential for maintaining telomere length in proliferating cells [1]. Because short telomeres impair cellular proliferative and regenerative capacity, TBDs can affect many organ systems, with hematologic, pulmonary, and hepatic manifestations among the most prominent in adults [3,4]. In the hematopoietic system, progressive loss of replicative capacity can lead to bone marrow failure and cytopenias, while impaired tissue regeneration can contribute to pulmonary fibrosis and increase susceptibility to hepatic injury [3-5].

In adults, the heterogeneous and often organ-predominant presentation can make TBDs challenging to diagnose, as hematologic, pulmonary, and hepatic manifestations may emerge at different times or initially appear unrelated [4]. In a recent adult TBD registry cohort, the median age at first clinical manifestation was 20.0 years, whereas the median age at diagnosis was 34.1 years, emphasizing the diagnostic delay that can be observed [6]. A suggestive personal and family history, together with telomere-length testing and germline genetic analysis, can help establish the diagnosis [3,4].

Heterozygous disease-associated variants in TERT are among the most common genetic causes of adult-onset TBD and are associated with incomplete, age-dependent penetrance and marked phenotypic variability, even within families [3]. Liver disease associated with TERT mutation may be misattributed to metabolic or toxic etiologies, although telomere dysfunction can lower the threshold for injury and accelerate fibrosis progression [5].

Danazol is a synthetic androgen with antigonadotropic and anabolic effects that is approved for the treatment of endometriosis and the prevention of hereditary angioedema attacks [7]. Androgens have also historically been used in bone marrow failure syndromes, and danazol has been repurposed for telomere diseases based on evidence that sex-hormone signaling can increase TERT expression and telomerase activity in hematopoietic cells [8,9]. In a prospective study of patients with telomere diseases, danazol produced hematologic responses in 79% of evaluable patients at 3 months and 83% at 24 months, and 92% of evaluable patients demonstrated telomere elongation at 24 months; however, liver enzyme elevations occurred in 41% [9]. Liver transplantation may treat end-stage hepatic disease in carefully selected patients with TBDs but does not directly treat the extrahepatic bone marrow disorder; consequently, marrow failure and other systemic manifestations may persist after transplantation, particularly under immunosuppression [10,11].

We present a case of a 45-year-old man with long-standing macrocytic pancytopenia and progressive cirrhosis who was ultimately diagnosed with a TERT-related TBD, treated with danazol, and later underwent orthotopic liver transplantation with concomitant sleeve gastrectomy.

## Case presentation

A 45-year-old man with a body mass index (BMI) of 65.8 kg/m² (reference: 19.5-24.5 kg/m²) was referred to a tertiary center for evaluation of long-standing macrocytic anemia, thrombocytopenia, and neutropenia since the age of 25. The patient had a history of obstructive sleep apnea (on bilevel positive airway pressure) and nonalcoholic fatty liver disease. The patient denied tobacco use and reported current alcohol intake of a few drinks per week but acknowledged heavy alcohol consumption during adolescence and early adulthood. Family history was notable for a mother diagnosed with myelodysplastic syndrome at 32 years of age, who later died of nonalcoholic cirrhosis at 57 years of age, and a sister who was recently found to have pancytopenia. Additional maternal relatives had early-onset cirrhosis, and there was a reported clustering of hematologic disorders and premature graying in the extended family.

Upon initial evaluation, the patient presented with macrocytic anemia, neutropenia, and thrombocytopenia, without requiring transfusions. Urinalysis was unrevealing with no proteinuria or hematuria. Bone marrow biopsy demonstrated mildly hypocellular marrow (approximately 35-40% cellularity) with trilineage hematopoiesis, no excess blasts, and normal cytogenetics. Additional testing revealed no paroxysmal nocturnal hemoglobinuria clone. Autoimmune serologies revealed an elevated anti-double-stranded DNA titer of 1:320, an elevated anti-nuclear antibody (ANA) with a 1:160 speckled pattern, and a low complement C4 level of 11 mg/dL (reference: 15-45 mg/dL); Sjögren antibodies were detected (unknown subtype).

Liver evaluation included an ultrasound that showed hepatomegaly with steatosis. Liver biopsy showed steatohepatitis, stage 2 periportal fibrosis, and increased copper deposition, interpreted as nonalcoholic steatohepatitis (NASH) on a background of metabolic risk factors. Serum ceruloplasmin was evaluated and was found to be normal at 30 mg/dL (reference: 19-42 mg/dL), along with a 24-hour urine copper measurement of 35 µg (reference: ≤60 µg/24 hours), findings that did not support Wilson disease in the setting of high hepatic copper deposition. Iron studies revealed a high transferrin saturation of 79% (reference: 20-50%), and an elevated ferritin at 458 ng/mL (reference: 30-400 ng/mL), but genetic testing for hereditary hemochromatosis was not diagnostic.

The combination of long-standing macrocytic pancytopenia, mildly hypocellular marrow without dysplasia, and family history of early-onset hematologic and hepatic disease with premature graying raised concern for an inherited telomere disorder, prompting referral to a national bone marrow failure center for further evaluation. Telomere length testing demonstrated markedly short telomeres in both lymphocytes (3.4 kb) and granulocytes (5.2 kb), each below the age-adjusted first percentile. An inherited bone marrow failure panel using next-generation sequencing (NGS) identified a heterozygous TERT c.2836T>C variant and a heterozygous Fanconi anemia complementation group A (FANCA) c.1874G>C variant. At the time of evaluation, both were described as pathogenic sequences in the available National Institutes of Health (NIH) record. These findings, together with the clinical phenotype and family history, supported the diagnosis of a TERT-related TBD.

Following diagnosis, the patient enrolled in a danazol trial in mid-2018 (400 mg/day orally, reduced to 200 mg/day orally after six months per protocol) and completed one year of therapy. Through this course, the patient’s hemoglobin increased from 10.0 to 13.8 g/dL (reference: 13.5-17.5 g/dL), and platelets rose from 53 to 142 ×10³/µL (reference: 150-450 ×10³/µL), representing a clear hematologic response.

In mid-2019, danazol was discontinued as the patient’s liver tests worsened and bilirubin rose. Outpatient laboratories later that year showed progressive cholestatic liver dysfunction with rising bilirubin and alkaline phosphatase, and declining albumin (Table 1). Right upper quadrant ultrasonography in November 2019 demonstrated volume redistribution, heterogeneous and coarsened hepatic echotexture, and nodular contour consistent with cirrhosis. Esophagogastroduodenoscopy (EGD) revealed grade I esophageal varices and portal hypertensive gastropathy. The patient was subsequently started on ursodiol (300 mg twice/day orally) and torsemide (20 mg twice/day orally).

**Table 1:** Longitudinal laboratory trends from initial presentation through danazol therapy, pre-transplant hepatic decompensation, and post-transplant follow-up MCV: mean corpuscular volume; WBC: white blood cells; AST: aspartate aminotransferase; ALT: alanine aminotransferase; INR: International Normalized Ratio

Parameter	November 2017 (initial evaluation)	April 2018 (pre-danazol treatment)	May 2019 (during danazol treatment)	October 2019 (post-danazol treatment)	September 2020 (Pre-transplant)	January 2021 (post-transplant)	2022-2025 (follow-up)	Reference range
Hemoglobin (g/dL)	10.4	10.0	13.8	10.3	8.7	9.5	10.9-13.0	13.5-17.5
MCV (fL)	123.4	125.1	119.4	125.3	109.3	105	104.9-116.4	80-100
WBC (10³/µL)	3.07	2.85	4.71	4.80	4.74	3.3	4.19-7.37	4-11
Platelet count (×10³/µL)	73	53	142	94	22	48	73-114	150-450
Total bilirubin (mg/dL)	0.8	0.9	2.5	6.2	10.4	0.8	0.4-0.7	0.2-1.3
Direct bilirubin (mg/dL)	0.3	0.4	1.3	4.0	6.5	0.3	0.1-0.3	0.0-0.5
AST (U/L)	35	42	80	114	611	16	14-21	10-50
ALT (U/L)	27	36	96	67	496	15	13-20	10-50
Alkaline phosphatase (U/L)	103	102	121	297	84	77	61-74	40-129
Albumin (g/dL)	3.5	3.3	3.9	2.6	3.0	4.3	4.1-4.5	3.5-5.2
INR	1.04	—	—	1.10	1.42	1.01	—	0.9-1.2
Creatinine (mg/dL)	1.06	0.90	1.15	1.00	1.76	1.73	1.24-1.57	0.73-1.18
Sodium (mmol/L)	141	140	138	139	143	143	137-143	136-145

In early 2020, the patient was hospitalized with volume overload requiring the addition of spironolactone to the medication regimen, followed by a later admission for hepatic encephalopathy in the setting of hyperammonemia. During the pre-transplant period, the patient’s MELD-Na (Model of End-Stage Liver Disease-Sodium) score was approximately 25 (total bilirubin 10.4 mg/dL, creatinine 1.76 mg/dL, International Normalized Ratio, or INR, 1.42, and sodium 143 mmol/L), with a Child-Pugh classification of C (indicating severe hepatic dysfunction based on bilirubin, albumin, INR, ascites, and hepatic encephalopathy). The patient was subsequently listed for liver transplantation and lost approximately 150 pounds with intensive dietary interventions, reducing the patient’s BMI from 65.8 to 41.0 kg/m² (reference: 19.5-24.5 kg/m²). Pre-operative workup included pulmonary evaluation with pulmonary function testing (PFT) that revealed normal spirometry and lung volumes but a moderately reduced diffusing capacity for carbon monoxide (DLCO 59%; hemoglobin-adjusted DLCO 77%), decreased from prior testing. A CT scan of the chest showed no pulmonary infiltrates or fibrosis. This raised concern for early interstitial lung disease, with possible contribution from TBD or autoimmune disease, while also considering portopulmonary hypertension or hepatopulmonary syndrome relating to cirrhosis and portal hypertension.

**Figure 1:**
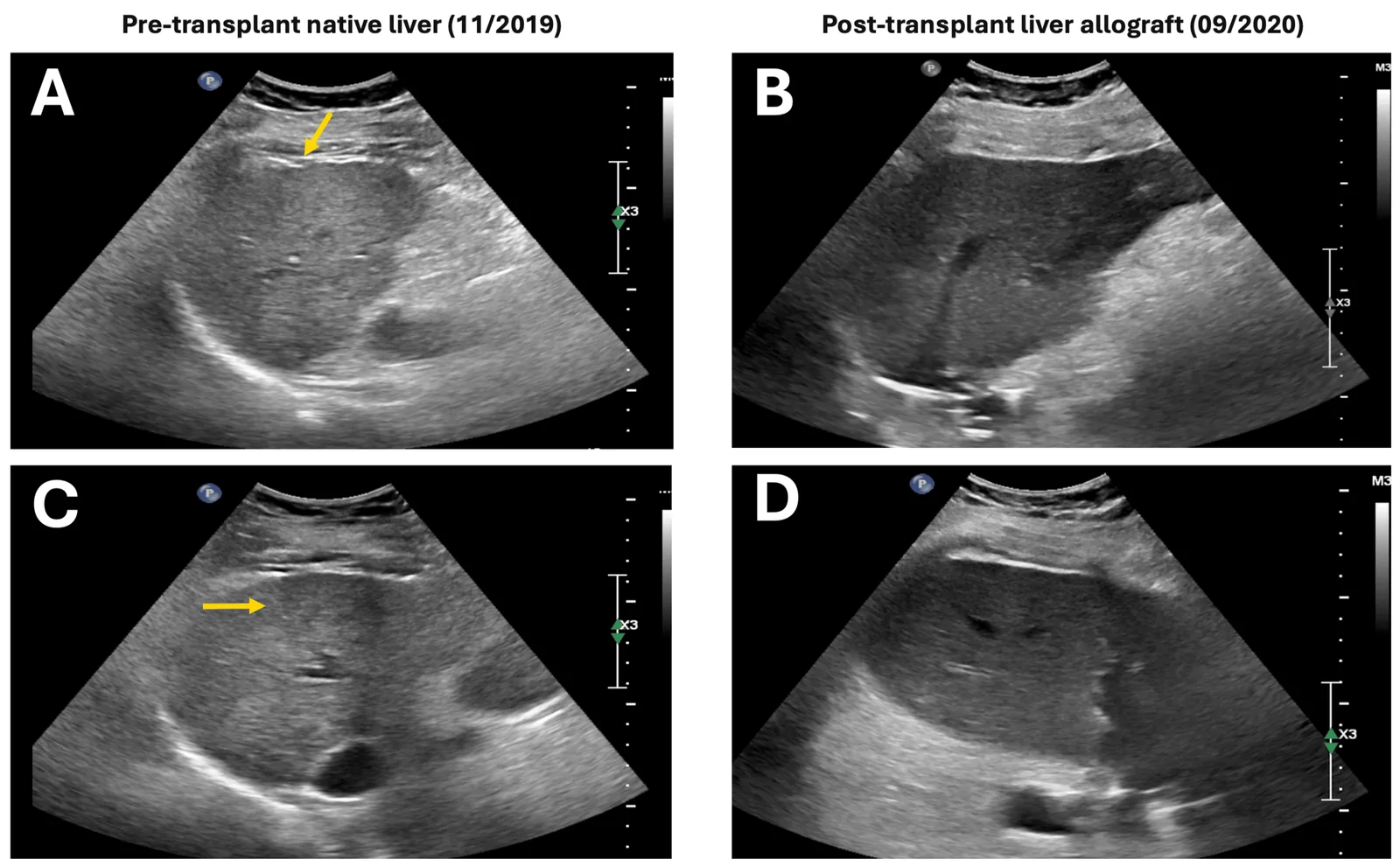
Right upper quadrant ultrasound of the pre-transplant cirrhotic native liver and post-transplant hepatic allograft (A) Pre-transplant longitudinal view of the native liver demonstrating nodular hepatic contour (arrow). (B) Post-transplant longitudinal view of the hepatic allograft demonstrating unremarkable morphology, parenchyma, and contour. (C) Pre-transplant transverse view demonstrating heterogeneous, coarsened hepatic parenchyma (arrow). (D) Post-transplant transverse view of the hepatic allograft demonstrating unremarkable morphology, parenchyma, and contour.

The patient underwent liver transplantation with concomitant sleeve gastrectomy in September 2020. Explant pathology confirmed cirrhosis with steatohepatitis and prominent hepatocellular iron deposition, without reported features suggestive of an autoimmune hepatitis pattern. Post-operatively, the patient received tacrolimus-based immunosuppression along with standard prophylactic medications. The early post-transplant course was notable for a gradual decline in blood counts. By late 2020, the patient required transfusion of two units of packed red blood cells (RBCs) for a hemoglobin of 6 g/dL (reference: 13.5-17.5 g/dL). A CBC approximately three months after transplant showed a white blood cell count of 3300/µL (reference: 4000-11000 /µL), a hemoglobin level of 9.5 g/dL (reference: 13.5-17.5 g/dL), a mean corpuscular volume of 105 fL(reference: 80-100 fL), and a platelet count of 48 ×10³/µL (reference: 150-450 ×10³/µL). Liver chemistries at that time were completely normalized (Table 1), with total bilirubin at 0.8 mg/dL (reference: 0.2-1.3 mg/dL), alkaline phosphatase at 77 U/L (reference: 40-129 U/L), aspartate aminotransferase at 16 U/L (reference: 0-35 U/L), and alanine aminotransferase at 15 U/L (reference: 0-45 U/L). Follow-up pulmonary testing again showed normal flows and total lung capacity with a persistent moderate diffusion defect and a significant decline in DLCO compared to previous testing; yearly repeat PFT and CT imaging were recommended. Post-transplant Doppler ultrasonography in September 2020 demonstrated unremarkable hepatic allograft morphology, parenchyma, and contour, with no focal lesion identified. Figure 1 compares the pre-transplant cirrhotic native liver with the post-transplant hepatic allograft.

**Figure 2:**
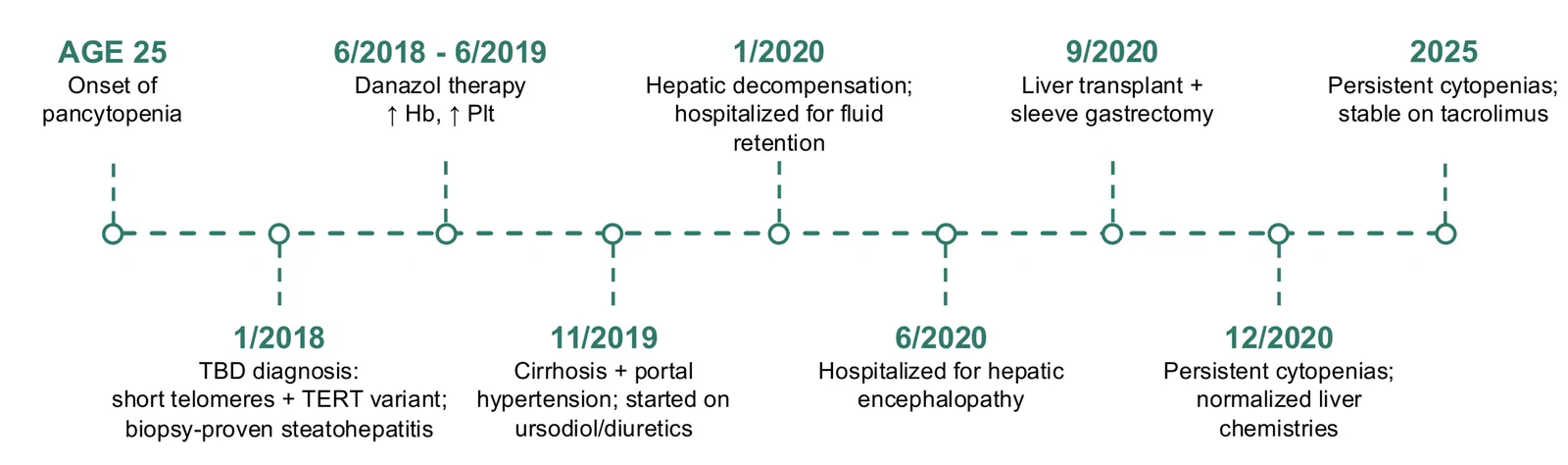
Clinical timeline of the patient’s course depicting key hematologic, hepatic, and therapeutic milestones from 25 years of age through 2025, including the onset of chronic cytopenias, diagnosis of TBD, initiation and discontinuation of danazol therapy, progression to hepatic decompensation, liver transplantation, and post-transplant persistence of cytopenias TBD: telomere biology disorder; TERT: telomerase reverse transcriptase; Hb: hemoglobin; Plt: platelets

On the most recent PFT in 2023, DLCO had improved to 76% predicted (83% hemoglobin-corrected), characterized as only mildly reduced, with normal spirometry and no bronchodilator response. In 2025, the patient remained stable on tacrolimus and was on a weight loss program, following up on an outpatient basis with Internal Medicine. The major hematologic, hepatic, and therapeutic milestones are summarized in Figure 2.

## Discussion

In our patient, the combination of long-standing macrocytic pancytopenia, markedly short telomeres, and a heterozygous TERT variant supports a systemic TBD. His cirrhosis was likely multifactorial rather than attributable solely to NASH or alcohol-related liver disease, while the coexisting bone marrow dysfunction is consistent with the multisystem phenotype of adult TBDs [3,4]. The patient’s biopsy-proven steatohepatitis with subsequent progression to cirrhosis and portal hypertension most likely reflects a liver disease in which telomere dysfunction may have lowered the threshold for injury and contributed to faster progression of the hepatic involvement. The pattern of myelodysplastic features, pancytopenia, early-onset cirrhosis, and premature graying among maternal relatives is consistent with the variable expressivity and age-dependent penetrance described in TERT-associated TBDs [3,4], further supporting a heritable telomere-mediated process superimposed on metabolic and toxic hepatic risk factors.

Autoimmune serologies and low complement raised the possibility of an overlapping autoimmune disease process. However, a diagnosis of connective tissue disease, including systemic lupus erythematosus (SLE) and Sjögren's syndrome, was not confirmed in available records. Hepatobiliary abnormalities have been reported in patients with both SLE and Sjögren's syndrome, primarily with nonalcoholic fatty liver disease and through overlap with other autoimmune diseases such as primary biliary cholangitis [12-14]. Concurrently, interstitial lung disease is a commonly recognized and often under-detected complication in both autoimmune diseases, which can manifest as decreased DLCO before radiographic involvement in its early form [15,16]. Thus, autoimmune overlap remained a consideration alongside the TBD-mediated liver and lung vulnerability.

Danazol led to clear hematologic improvement, as shown by the increase in hemoglobin and platelet counts following the start of therapy. However, worsening cholestasis occurred during the period surrounding androgen therapy, and hyperbilirubinemia persisted after danazol was discontinued. Although causality cannot be established, there was a temporal association between androgen exposure and worsening cholestasis. Given the potential for androgenic steroids such as danazol to cause cholestatic liver injury [17], close hepatic monitoring and repeated assessment of the risk-benefit balance are warranted when danazol is used in patients with underlying liver disease [9,17].

Pulmonary evaluation showed no radiographic fibrosis on CT, but a persistent moderate diffusion defect and declining DLCO on PFT, raising concern for possible subclinical pulmonary involvement in the setting of TERT-related disease. This supports the idea that organ involvement in TBDs can be present before obvious imaging abnormalities and that multi-organ assessment is essential when considering liver transplantation in TERT-related disease [4,10]. On interval testing in 2023, DLCO improved from 59% to 76% predicted but remained below the normal range, characterized as mildly rather than moderately reduced. This partial improvement may reflect resolution of cirrhosis-related and obesity-related contributors to the diffusion defect, such as hepatopulmonary or portopulmonary physiology and the patient's substantial post-transplant weight loss, rather than resolution of an underlying telomere-mediated process; the persistence of a mild defect may be consistent with ongoing subclinical pulmonary involvement. Continued pulmonary follow-up is warranted as TERT-related pulmonary fibrosis can progress rapidly [18].

Liver transplantation and sleeve gastrectomy resulted in normalization of liver chemistries and significant weight loss, but cytopenia recurred under tacrolimus-driven immunosuppression. This emphasizes that the transplant corrected the hepatic consequences of telomere failure but not the underlying marrow disorder, and it highlights the need for ongoing hematology, hepatology, rheumatology, and pulmonary follow-up [4,18].

An additional consideration is the changing interpretation of the patient’s genetic findings. At the time of diagnosis, the NIH records described both the TERT c.2836T>C and FANCA c.1874G>C variants as pathogenic sequences. The original molecular laboratory report containing formal American College of Medical Genetics and Genomics (ACMG) classification criteria was not available for review. ClinVar, a publicly accessible National Center for Biotechnology Information (NCBI) archive of submitted clinical variant interpretations, currently lists the TERT variant as a variant of uncertain significance (VUS), while the FANCA variant has conflicting classifications ranging from VUS to benign/likely benign [19,20]. Therefore, the current classification introduces uncertainty regarding the specific contribution of the TERT variant. However, its occurrence in a patient with telomere lengths below the first percentile in both lymphocytes and granulocytes, long-standing macrocytic pancytopenia, and a suggestive family history raises the possibility that the variant may still be clinically relevant. This case cannot establish its pathogenicity directly but provides additional phenotypic context for future evaluation of this TERT variant.

Given this multifactorial picture, the relative contribution of telomere dysfunction to this patient’s hepatic outcome cannot be directly established, and autoimmune serologies raised the possibility of an overlapping connective tissue disease that was never definitively confirmed. Although pulmonary involvement was demonstrated by a persistent diffusion defect, the absence of radiographic pulmonary fibrosis limited characterization of the specific pulmonary phenotype and the relative contributions of telomere-mediated disease.

## Conclusions

This case of a TERT-related TBD presenting with long-standing macrocytic pancytopenia and cirrhosis with progressive hepatic decompensation illustrates the coexistence of abnormal telomere biology with metabolic and toxic hepatic risk factors in a patient who ultimately developed hepatic decompensation. Danazol therapy produced a clear hematologic response, while worsening cholestasis occurred in temporal association with androgen exposure without an established causal relationship. Liver transplantation with sleeve gastrectomy resulted in good hepatic and metabolic outcomes, but extrahepatic manifestations persisted, including recurrent cytopenias and a residual pulmonary diffusion defect, and autoimmune overlap remained a diagnostic consideration given the patient's serologic profile. The current listing of the TERT c.2836T>C variant as VUS also illustrates the evolving nature of genetic interpretation and the importance of considering variant classification alongside telomere length, clinical phenotype, and family history. Clinicians should maintain a high index of suspicion for TBD in adults with unexplained cytopenias and concomitant liver disease and integrate telomere testing and genetic panels into the diagnostic work-up.
